# A cDNA Immunization Strategy to Generate Nanobodies against Membrane Proteins in Native Conformation

**DOI:** 10.3389/fimmu.2017.01989

**Published:** 2018-01-23

**Authors:** Thomas Eden, Stephan Menzel, Janusz Wesolowski, Philine Bergmann, Marion Nissen, Gudrun Dubberke, Fabienne Seyfried, Birte Albrecht, Friedrich Haag, Friedrich Koch-Nolte

**Affiliations:** ^1^Institute of Immunology, University Medical Center Hamburg-Eppendorf, Hamburg, Germany

**Keywords:** nanobody, heavy-chain antibody, antibody engineering, cDNA immunization, genetic immunization, membrane proteins

## Abstract

Nanobodies (Nbs) are soluble, versatile, single-domain binding modules derived from the VHH variable domain of heavy-chain antibodies naturally occurring in camelids. Nbs hold huge promise as novel therapeutic biologics. Membrane proteins are among the most interesting targets for therapeutic Nbs because they are accessible to systemically injected biologics. In order to be effective, therapeutic Nbs must recognize their target membrane protein in native conformation. However, raising Nbs against membrane proteins in native conformation can pose a formidable challenge since membrane proteins typically contain one or more hydrophobic transmembrane regions and, therefore, are difficult to purify in native conformation. Here, we describe a highly efficient genetic immunization strategy that circumvents these difficulties by driving expression of the target membrane protein in native conformation by cells of the immunized camelid. The strategy encompasses ballistic transfection of skin cells with cDNA expression plasmids encoding one or more orthologs of the membrane protein of interest and, optionally, other costimulatory proteins. The plasmid is coated onto 1 µm gold particles that are then injected into the shaved and depilated skin of the camelid. A gene gun delivers a helium pulse that accelerates the DNA-coated particles to a velocity sufficient to penetrate through multiple layers of cells in the skin. This results in the exposure of the extracellular domains of the membrane protein on the cell surface of transfected cells. Repeated immunization drives somatic hypermutation and affinity maturation of target-specific heavy-chain antibodies. The VHH/Nb coding region is PCR-amplified from B cells obtained from peripheral blood or a lymph node biopsy. Specific Nbs are selected by phage display or by screening of Nb-based heavy-chain antibodies expressed as secretory proteins in transfected HEK cells. Using this strategy, we have successfully generated agonistic and antagonistic Nbs against several cell surface ecto-enzymes and ligand-gated ion channels.

## Introduction

**Figure 1:**
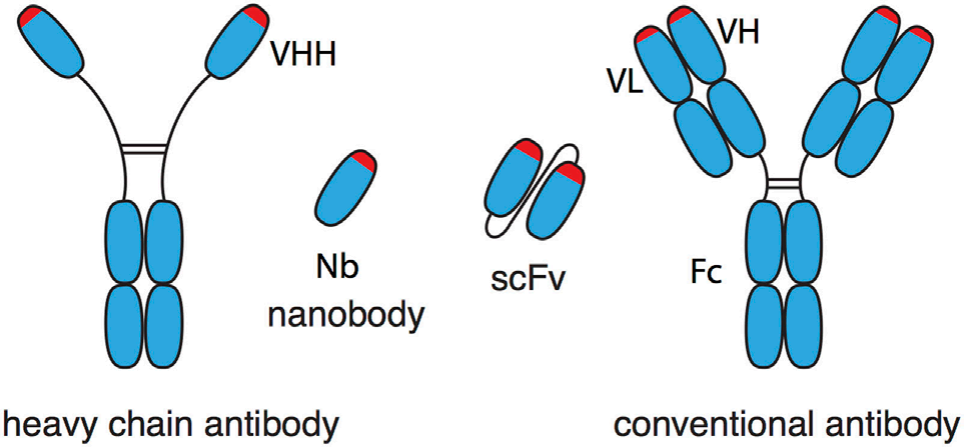
Schematic comparison of nanobodies (Nbs) from heavy-chain antibodies and single-chain variable fragments (scFv) from conventional antibodies. Nbs correspond to the variable domain (VHH) of heavy-chain antibodies. Nbs generally show much better solubility and stability than the corresponding pair of variable domains (VH, VL) of conventional antibodies, even when the latter are connected by a synthetic peptide linker into a scFv.

Nanobodies (Nbs), single-domain antibodies derived from camelid heavy-chain antibodies, are 10-fold smaller than conventional antibodies (Figure 1) and exhibit a number of advantageous features (1–4). In Nbs, the core of the antigen recognition site, i.e., the CDR3 loop, is often much longer than that of conventional antibodies. Consequently, Nbs can bind epitopes that are inaccessible to conventional antibodies, e.g., cryptic functional epitopes such as the active site of an enzyme, the ligand-binding site of an ion channel, or the virus-binding site of a cell surface receptor (5). The single-domain format of Nbs greatly facilitates the construction of multi-specific and multivalent biologics by genetically linking Nbs in a linear fashion. Genetic fusion can endow Nbs with additional effector functions, e.g., cytotoxicity, extended *in vivo* half-life, or translocation through the blood–brain barrier (6–9). Moreover, as chaperones in protein crystallography, Nbs can greatly aid structure function analyses (10, 11). *In vivo*, monomeric Nbs penetrate tissues better than conventional antibodies. Importantly, the *in vivo* half-life of Nbs can easily be adjusted, e.g., by genetic fusion to an albumin-specific Nb (6). To date more than 1,000 patients and healthy subjects have received Nbs in clinical studies without any obvious off-target side effects or the induction of neutralizing antibodies. Caplacizumab is the first Nb for therapy expected to receive approval for the clinic in 2018 (12).

Membrane proteins are particularly interesting Nb targets in research, diagnosis, and therapy (2, 3, 13–15). The extracellular domain(s) of a membrane protein often contain(s) several epitopes accessible to Nbs. Nbs targeting such epitopes can be converted into effective tools for structural studies and for visualizing and tracing membrane proteins on living cells, e.g., by high resolution microscopy (16, 17). Nbs can be used also to mark cells for sorting by flow cytometry or magnetic beads. Antagonistic or agonistic Nbs can be used to modulate the function of the membrane protein and/or the cell expressing the membrane protein. In case of cancer cells, opsonization with Nb-based heavy-chain antibodies can aid anti-tumor responses. Moreover, Nbs can be used to deliver imaging agents, cytotoxic compounds, and immune cells to tumor cells expressing the target membrane protein *in vivo*.

For such experimental and therapeutic applications, it is important that the Nb recognizes its target membrane protein in native conformation. However, raising Nbs against a membrane protein of interest in native conformation can be challenging. Antibodies induced by synthetic peptides usually work well in Western Blot analyses, where the membrane protein is denatured. Anti-peptide antibodies, however, usually fail to recognize the natively folded protein on the cell surface of living cells. Genetic fusion of peptides to a multimerization domain has been shown to enhance the chance of inducing Nbs against native proteins (12). Purification of membrane proteins for immunization is often hampered by the necessity to use detergents and the tendency of membrane proteins to aggregate upon removal of the detergent (16). Numerous strategies have been employed successfully to overcome these hurdles, including immunization with transfected cells overexpressing the membrane protein of interest and incorporation of the purified membrane protein into liposomes or into the outer membrane of an enveloped virus (14, 18). Notwithstanding, other proteins present in such preparations can interfere with the immune response against the membrane protein of interest.

Genetic or cDNA immunization poses an attractive alternative (19). This strategy aims to transfect cells of the host animal with a mammalian expression vector, akin to transfection of HEK cells in cell culture. The goal of the cDNA immunization strategy is to drive faithful expression and posttranslational modification of the membrane protein of interest on the plasma membrane of skin and immune cells of the immunized animal. Here, we describe a cDNA immunization strategy that has allowed us to generate Nbs and Nb-based heavy-chain antibodies against membrane proteins from immunized llamas (8, 20–22). Moreover, we discuss some of the pitfalls and options for adapting this strategy to other membrane proteins.

## Membrane Proteins as Targets for Nbs

Membrane proteins are interesting targets for therapeutic Nbs because membrane proteins are accessible to systemically injected biologics. For example, Nbs directed against membrane proteins on immune cells may provide an effective means to enhance or dampen immune responses (20–23). Furthermore, Nbs directed against membrane proteins specifically expressed by cancer cells represent potential diagnostics and therapeutics (1, 8, 24–30).

**Figure 2:**
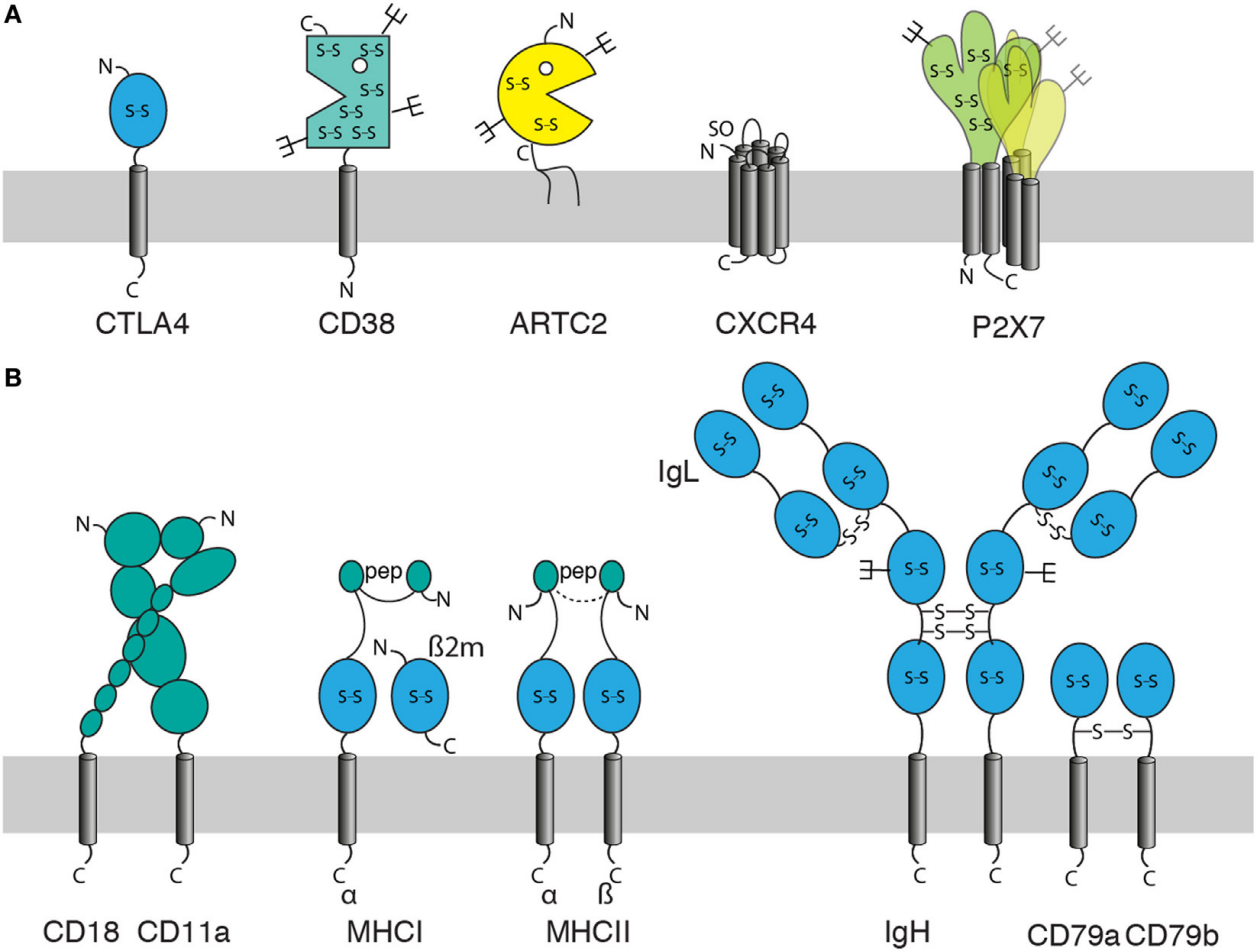
Features of membrane proteins to consider for generation of nanobodies (Nbs) by the cDNA immunization strategy. When using cDNA immunization as a strategy to induce a Nb response against a membrane protein, it is important to consider: the size and structure of its extracellular domain(s), the location of its N- and C-termini (out, in), posttranslational modifications [glycosylation, sulfation], and whether the protein can be expressed alone or in association with partner proteins only. Posttranslational modifications include glycosylation (forks), disulfide bridges (S-S), sulfation (SO), and loading with peptide (pep). **(A)** Examples of monomeric and homo-multimeric membrane proteins: GPI-anchored proteins such as ARTC2 consist of an extracellular domain covalently linked *via* the C-terminal amino acid to a membrane glycolipid. Single span membrane proteins possess extracellular and intracellular domains (or chains of linked domains). The extracellular domain is N-terminal in type I membrane proteins such as CTLA-4 and C-terminal in type II membrane proteins such as CD38. Most double-spanning (and tetra-spanning) membrane proteins have cytosolic N- and C-termini. Some double-spanning proteins such as P2X7 exist as homomultimers. Seven transmembrane proteins [G-protein-coupled receptors (GPCRs)] such as CXCR4 have an N-out C-in orientation and typically exist as monomers or dimers. **(B)** Effective expression of multimeric membrane proteins on the cell surface may require co-expression of one or more partners. These can be other transmembrane, secretory, or cytosolic proteins. Integrins such as LFA-1 (CD11a/CD18) are efficiently expressed on the cell surface only as a pair of non-covalently linked type I membrane proteins. MHC class I molecules are composed of a type I membrane protein, a non-covalently associated secretory protein (β2m) and a peptide docked in the peptide binding groove, MHC class II molecules are composed of two non-covalently linked type I proteins and a docked peptide. Many receptor complexes are assembled from three or more proteins, some of which may be linked by interchain disulfide bonds, as in the B cell receptor (BCR) complex where disulfide bridges link the two heavy chains (type I), each heavy chain to a light chain (secretory protein), and the two accessory type I proteins CD79a and CD79b.

When using cDNA immunization as a strategy to generate Nbs, it is important to consider the molecular architecture of the membrane protein of interest (Figure 2). Often, the extracellular domain is composed of a single well-defined protein domain: the checkpoint inhibitor CTLA-4 contains a single immunoglobulin domain, the ecto-enzymes CD38 and ARTC2 a single, well-defined catalytic domain (Figure 2A). Other proteins can carry two or more distinct extracellular protein domains, such as integrins or MHC proteins (Figure 2B). Membrane proteins with such large extracellular domains often offer many independent accessible epitopes for different Nbs. However, some membrane proteins, including many G-protein-coupled receptors (GPCRs) and voltage gated ion channels contain only very small extracellular domains encompassing only a handful of amino acid residues. Some of these membrane proteins may not offer sufficient space on the extracellular leaflet of the plasma membrane for even a single Nb.

When the membrane protein of interest is a monomer or homomultimer with a large ecto-domain (Figure 2A), cDNA immunization generally is rather straightforward since the protein does not require other proteins for stable expression on the cell surface in native conformation. However, many membrane proteins stably associate with one or more other proteins (Figure 2B). Stable heterodimers, for example, are formed by the alpha and beta chains of integrins and the alpha and beta chains of MHC molecules in association with a non-covalently bound peptide. Other membrane proteins such as the B cell receptor (BCR) complex are assembled into multimeric receptor complexes.

For membrane proteins that form heterodimeric or multimeric complexes with other membrane proteins, the cDNA immunization strategy may need to be adjusted. One option is to take the chance that the membrane protein of interest pairs with the orthologous partner protein(s) of the immunized host. For ubiquitously expressed proteins and for proteins expressed by skin cells or Langerhans cells, this may work. However, this is highly risky since the interphase surfaces of the pairing proteins might not be conserved. A better option is to immunize with a mixture of expression constructs encoding all members of the protein complex. This would require co-transfection and co-expression in individual cells. While this is highly efficient with a combination of two vectors, it may be less efficient with multiple vectors.

**Figure 3:**
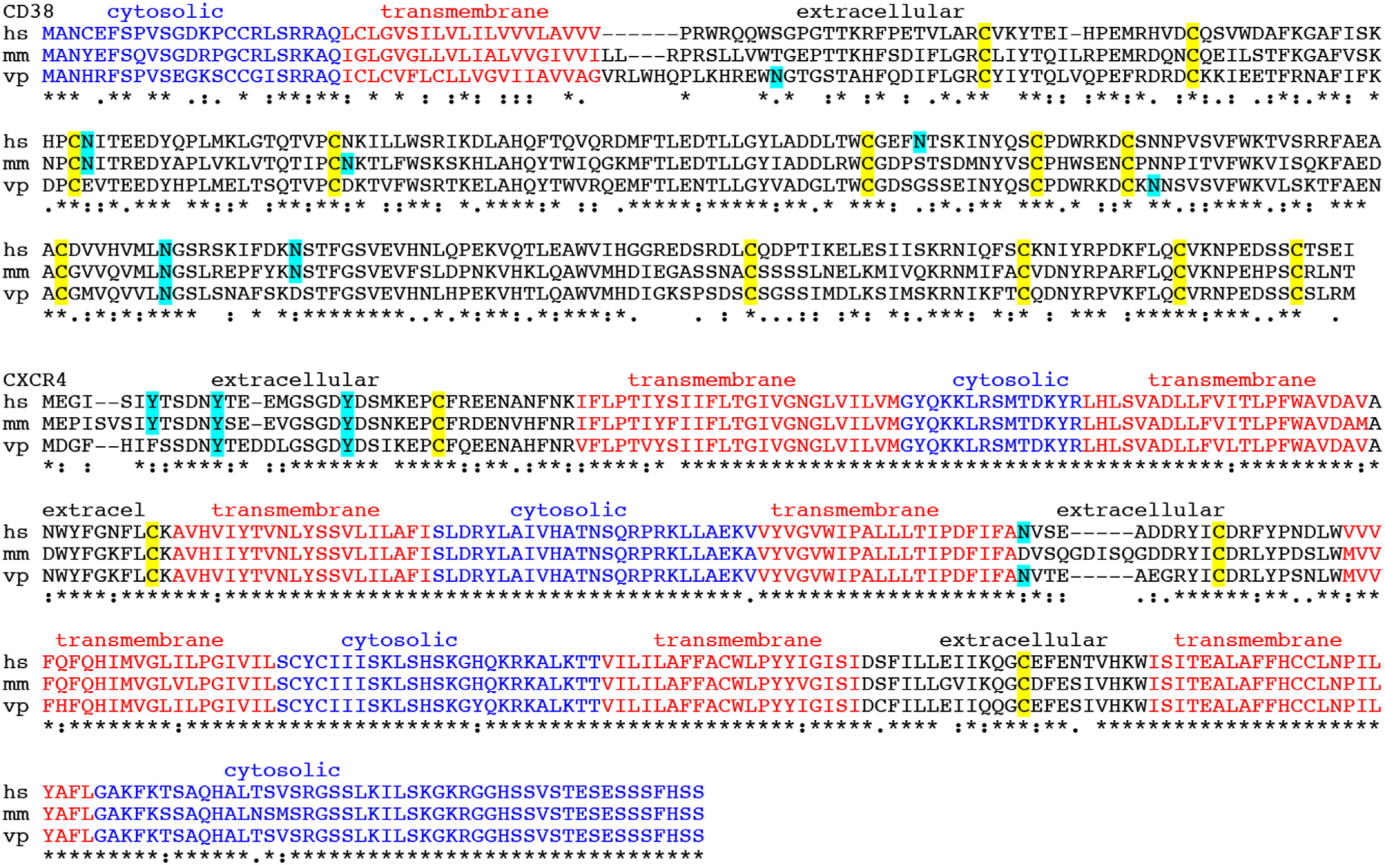
Amino acid sequence alignments of CD38 and CXCR4 from human, mouse, and alpaca. Amino acid sequence alignments of the CD38 and CXCR4 species orthologs from human, mouse and alpaca (hs, *homo sapiens*; mm, *mus musculus*; vp, *vicuna pacos*). Symbols below the alignments indicate identical amino acid residues (*), conserved substitutions (. and:), and non-conserved substitutions (blank spaces). Cytosolic amino acid residues are indicated in blue, transmembrane residues in red, and extracellular residues in black. Potential asparagine-linked glycosylation sites and potential tyrosine-linked sulfation (SO) sites are highlighted in cyan. Cysteines engaged in intrachain disulfide bonding are highlighted in yellow. In order to access the sequence of the camelid ortholog, we perform a protein BLAST search (https://blast.ncbi.nlm.nih.gov) using the human protein sequence as input and selecting “Non-redundant protein sequences (nr)” as database and “Camelidae (taxid:9835)” as organism from the respective pull down menus.

In addition to the molecular architecture of the membrane protein of interest, it is important to analyze the conservation of its amino acid sequence to the orthologous protein of the camelid since structural conservation can restrict the antibody response to particular epitopes. In extreme cases, i.e., for a highly conserved protein, it may even be impossible to raise antibodies in camelids. However, since camelids are outbred animals, a dromedary, bactrian camel, llama or alpaca might have a sufficiently diverse ortholog and/or a set of suitable MHC alleles, so that an antibody response can be induced. The immune response of a healthy animal usually ensures that antibodies induced by immunization recognize only those epitopes of the target protein that are different from its own species ortholog, thereby preventing auto-antibody responses. We, therefore, routinely perform an amino acid sequence alignment of the membrane protein of interest and its orthologs from human, mouse and llama (Figure 3). With the aid of such an alignment, it is often possible to predict whether known or potential posttranslational protein modifications, including glycosylation, SO, and disulfide bond formation, are conserved (Figure 3). If the 3D-structure of one or more species orthologs is known, the alignment may even permit the prediction of possible Nb binding epitopes. The sequence alignment also points out residues that are conserved in mouse and human but not in the camelid. These are potential targets for a cross-reactive antibody. However, it is impossible to predict whether such cross-reactive antibodies will actually be obtained.

In the case of CD38 only one of four N-linked glycosylation sites in the ecto-domain of human CD38 is conserved also in the alpaca homolog. Moreover the ecto-domains of human and alpaca CD38 differ in more than 50 amino acid residues. In the case of CXCR4, the extracellular loops of the human and alpaca orthologs differ by ~20 amino acid substitutions. Based on the degree of sequence conservation, it is more likely to obtain a heavy-chain antibody response in alpacas against CD38 than against CXCR4. Case studies in the literature indicate that antibody responses can be induced even for antigens that differ only in one or a few amino acid substitutions. In cases of high sequence conservation, tolerance may be overcome by co-immunizing with protein that can deliver foreign peptides to MHCI and MHCII molecules (see below, section on mechanism of antibody induction).

## Cloning and Validation of the cDNA Expression Vector Encoding the Membrane Protein of Interest

**Figure 4:**
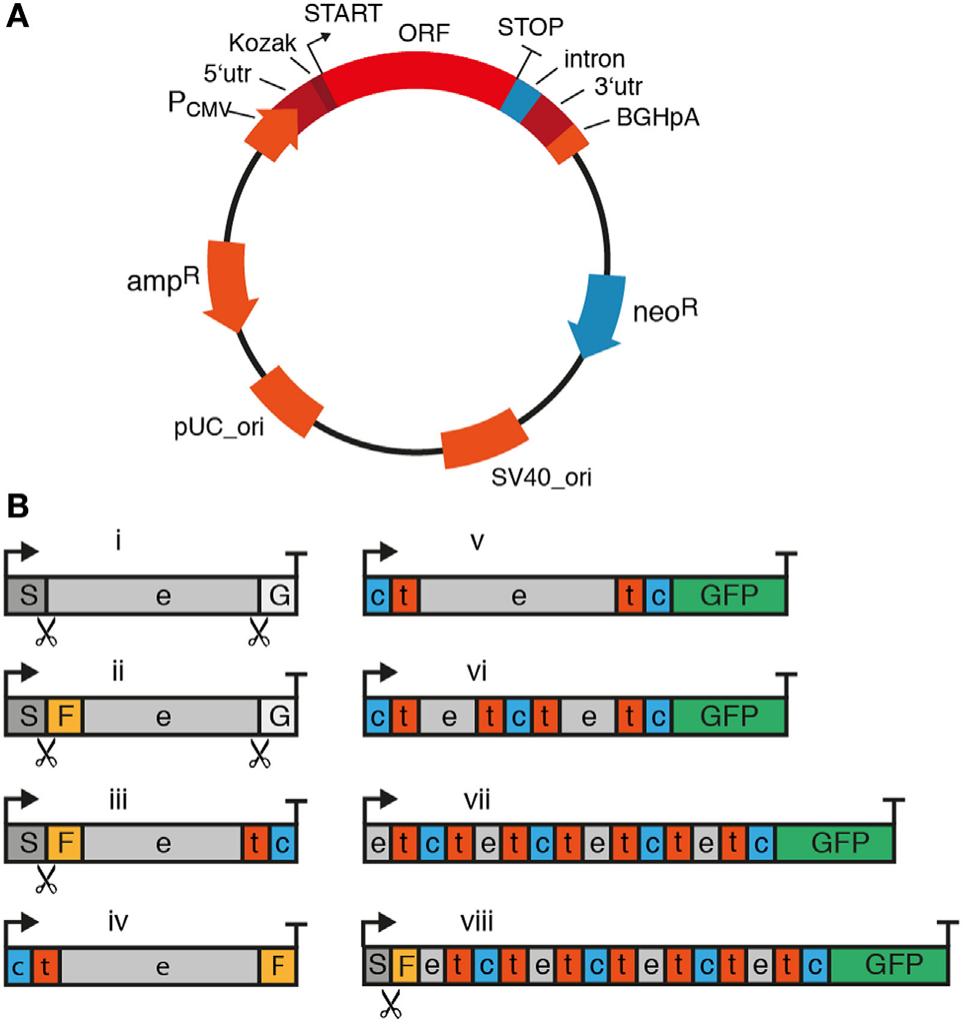
Essential and optional features of mammalian expression vectors for cDNA immunization of camelids. When using cDNA immunization as a strategy to induce a nanobody (Nb) response, it is important to consider components of the plasmid and the cDNA expression cassette. **(A)** Essential components of the plasmid are indicated in red and optional components in blue. The ampicillin resistance (ampR) and bacterial origin of replication (pUC_ori) are required for plasmid propagation in *E. coli*. The expression cassette for the membrane protein of interest contains the entire open reading frame (ORF) preceded by a strong viral promoter (e.g., the cytomegalovirus promoter, P_CMV_), a 5′ untranslated region (5′utr) and a Kozak sequence upstream of the start codon (arrow). The ORF is followed downstream of the stop codon by a 3′ untranslated region (3′utr) and a polyadenylation signal (e.g., of the bovine growth hormone, BGHpA). A mammalian antibiotic resistance gene (e.g., neomycin phosphotransferase, neoR) can be used for selecting stably transfected cells in culture. During cDNA immunization, this protein can help drive the immune response by providing peptides presented by MHC molecules. An intron can be placed anywhere between the start codon and the poly-adenylation signal to promote transcription and RNA processing. **(B)** In its simplest format (i), the expression cassette contains the full length open reading frame from start codon (arrow) to stop codon (T). A fused tag (e.g., Flag-tag, F) or fluorescent protein [e.g., green fluorescent protein (GFP)] can provide a means to verify expression of the protein at the plasma membrane. For fusion to a tag or protein, it is important to consider the availability and localization of the N- and C-termini of the membrane protein. Many membrane proteins contain a cleaved signal sequence (S) at the N-terminus, i.e., type I membrane proteins (iii) and some G-protein-coupled receptors (GPCRs) (viii). GPI-anchored proteins (i, ii) additionally contain a C-terminal signal sequence (G). The cleavage sites for these signal sequences are indicated by scissors. GFP usually works best when attached to a cytosolic terminus (v–viii). e: extracellular, t: transmembrane, c: cytosolic.

The cDNA expression vectors used for genetic immunization are classical vectors used also for transfection of HEK cells or CHO cells, such as pCMV-Sport6 or pCDNA3 (Invitrogen). The sequences of these vectors are publicly available in the online repository of addgene (https://www.addgene.org/vector-database). Figure 4 summarizes the essential and some optional features of a suitable cDNA expression vector. Essential components include the open reading frame (ORF) encoding the membrane protein of interest, a strong ubiquitous promoter, and a bacterial antibiotic resistance gene. Optional features include a mammalian antibiotic resistance gene, epitope tags, and an intron. A strong ubiquitous promoter ensures transcription of the membrane protein’s open reading frame in cells of the immunized camelid. The promoter commonly is derived from a mammalian virus such as cytomegalovirus. A bacterial antibiotic resistance gene such as β lactamase (ampR) and an origin of replication (pUC_Ori) are required for plasmid propagation in *E. coli*. Since the ampR gene is under control of a bacterial promoter, it is not expressed by mammalian cells and, thus, not relevant for the *in vivo* immune response.

Many available expression vectors contain a second antibiotic resistance gene under control of a eukaryotic promoter, e.g., neomycin phosphotransferase II (neoR) mediating resistance to neomycin under control of the SV40 viral promoter (Figure 4A). This resistance marker provides the option of selecting stable transfectants in cell culture. Since this gene is under control of a eukaryotic promoter, it will be expressed by the transfected skin cells of the immunized camelid. Expression of the antibiotic resistance marker may actually enhance an adaptive immune response, since neomycin phosphotransferase can serve as a source of peptides for presentation by MHCI molecules and thereby amplify cytotoxic T cell responses. T cell-mediated cytolysis of transfected cells can facilitate uptake of vesicles containing the membrane protein by antigen-specific B cells (see Mechanism of Antibody Induction by Genetic Immunization).

In order to verify that the expression vector effectively drives the production of the target protein on the plasma membrane, it may be useful to genetically fuse a fluorescent protein (e.g., GFP) and/or a peptide tag (e.g., a FLAG-tag) to the protein of interest (Figure 4B). The fused GFP can also enhance the antibody response by providing foreign MHC-binding peptides as helper epitopes (see Figure 6). For this, it is important to consider the availability and localization of the N- and C-termini of the membrane protein. Many membrane proteins contain a cleaved N-terminal signal sequence and—in the case of GPI-anchored proteins—a C-terminal signal sequence as well. A fluorescent protein provides a means to verify expression of the fusion protein at the plasma membrane of transfected cells—GFP usually works best when attached to a cytosolic terminus. Similarly, an extracellular epitope-tag provides a means to verify cell surface expression of the membrane protein in transfected cells using a fluorochrome-conjugated tag-specific antibody in microscopy or flow cytometry. For GPI-anchored proteins, only the N-terminus is available for fusion since the C-terminus is covalently attached to a glycolipid. In this case—as with most type I membrane proteins that contain a cleavable signal sequence—the epitope-tag needs to be placed behind the signal sequence since this is proteolytically removed during translation in the ER.

## Coating of Plasmids to Gold Particles and Ballistic cDNA Immunization

Ballistic transfection of skin cells with plasmid-coated gold particles is a very efficient technique to transfect skin cells including epithelial cells (EC), endothelial cells, and professional antigen-presenting cells (APC; e.g., Langerhans cells) (19, 31, 32). Other techniques for cDNA transfection have been used successfully (33–35). Even the simple injection of plasmid DNA in saline can lead to transfection, albeit usually with lower efficiencies (36). High pressure or electric pulses can be used to enhance transfection efficiencies (34, 35, 37, 38).

**Figure 5:**
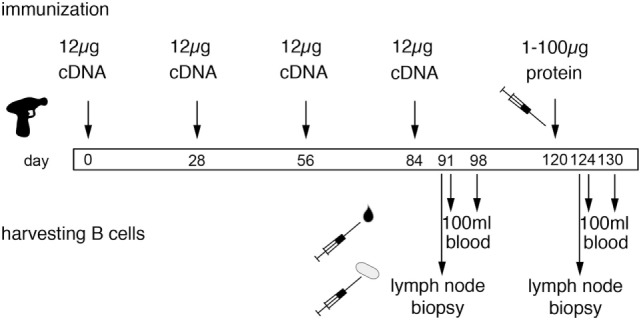
Schematic diagram of the genetic immunization strategy to generate nanobodies (Nbs) against membrane proteins. The scheme illustrates time points for immunization and harvesting of blood cells in order to obtain Nbs directed against membrane proteins in native conformation. Each cDNA immunization comprises 12 shots, each with 1 µg plasmid DNA coated onto 1 mg of gold particles (see Figure 4). Boost immunizations are spaced 3–4 weeks apart. A final protein boost is performed with the purified ecto-domain, cells transfected with the membrane protein of interest, or protein immunoprecipitated from transfected cells with bead-conjugated antibodies. B cells are harvested from a sample of blood and/or a lymph node biopsy 7 days after the last cDNA boost and 4 days after the last protein boost, i.e., at the peak of lymph node swelling. A second sample of blood is drawn 6–7 days thereafter. i.e., at the estimated peak of migration of expanded B cells from the lymph node to the bone marrow. We have selected antigen-specific Nbs from libraries constructed from lymphocytes obtained both before and after the protein boost. In case of similar Nbs obtained from both samplings, it can be inferred that the respective clone was induced by DNA immunization—and that this clone was probably reactivated by the final protein boost.

DNA immunization of larger animals has been problematic, possibly owing to the tougher texture of the skin. Our strategy to overcome this issue includes careful thorough shaving of the lower neck, followed by treatment with a commercial depilation cream, and use of gene gun-mediated ballistic immunization at a high pressure setting. In our experience, this approach is a highly efficient and reproducible technique to induce a heavy-chain antibody response in camelids (20–22). For ballistic cDNA immunization of camelids, we essentially follow the protocol of the provider of the Helios gene gun (BioRad). In brief, freshly purified plasmid DNA is precipitated onto spermidine-coated 1 µm gold particles using CaCl_2_. 50 µg ultrapure plasmid DNA coated onto 25 mg of gold particles provides sufficient material for 48 shots (4 rounds of immunization). The precipitate is washed and resuspended in absolute ethanol containing polyvinylpyrrolidone. The DNA-coated gold particles are dried onto the inner wall of a long plastic tubing with a gentle flow of nitrogen gas under gentle rotation of the tubing. The tubing with the dried gold particles is cut into 48 cartridges, 12 of which are loaded into the cartridge holder of a gene gun. The gene gun is connected to a helium flask *via* a pressure gauge set at 600 psi. The nozzle of the gene gun is placed approximately 1 cm above the shaved skin of the camelid. Pulling the trigger releases a helium pulse that accelerates the DNA-coated gold particles to a velocity sufficient to penetrate through multiple layers of cells in the skin and to penetrate the plasma membrane of cells in the trajectory. The skin at the site of injection is prepared for immunization by shaving and by depilation using a mild depilation cream. For camelids, the skin of the lower neck and that of the upper hind leg are suitable injection sites. Since the camelid skin is tougher than that of rabbits or rodents, we use a higher pressure setting for camelids (600 psi) than for rodents and rabbits (400 psi). We typically use 12 cartridges per immunization (1 µg plasmid DNA 0.5 mg gold particles per shot). Boost immunizations are performed every 2–6 weeks (Figure 5). Longer intervals may improve the antibody response by providing more time for affinity maturation but come at increased costs for animal housing.

**Figure 6:**
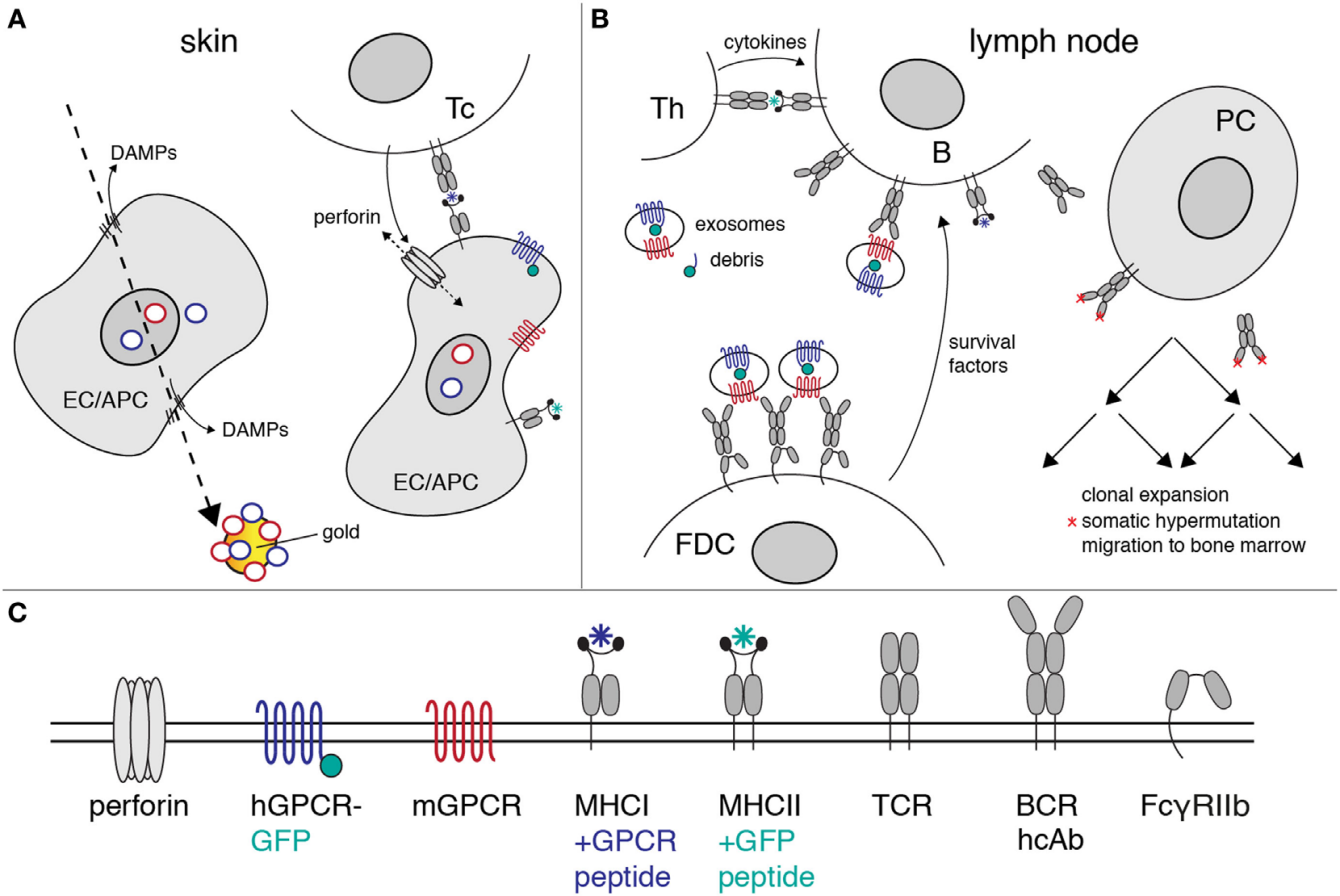
Proposed mechanism of heavy-chain antibody induction by genetic immunization. The figure illustrates the likely mechanism of heavy-chain antibody induction by genetic immunization of a camelid. **(A)** Ballistic injection of gold particles coated with cDNA expression vectors (rings) transfects some epithelial cells (EC) and antigen-presenting cells (APC) in the skin. Injury of the plasma membrane by the penetrating gold particles results in the release of danger-associated molecular patterns (DAMPs). Here, gold particles carry a cocktail of expression vectors encoding a mouse G-protein-coupled receptor (GPCR) (red) and its human homolog (blue) as a fusion protein to green fluorescent protein (green). Transcription and translation results in cell surface expression of native mouse and human GPCRs. Peptides derived from these proteins are presented by MHC-I molecules on the cell surface. A cytotoxic T cell (Tc) with a matching T cell receptor (TCR) is induced to secrete perforins. Insertion of perforins into the plasma membrane of the EC/APC results in lysis of the target cell. **(B)** Exosomes and cellular debris released during cell lysis are transported to the draining lymph nodes. B cells with a matching heavy-chain B cell receptor (BCR) endocytose the immunogen-containing exosomes and debris. Peptides derived from endocytosed proteins are presented on the B cell surface by MHC-II molecules. A helper T cell (Th) with a matching TCR releases growth-promoting cytokines. Follicular dendritic cells (FDCs) bind immune complexes *via* the low-affinity Fc receptor (FcγRIIb). FDCs present complexes of antibodies and immunogen to B cells expressing matching heavy-chain antibodies. Th cytokines and survival factors provided by FDC induce: (i) clonal expansion of B cells expressing specific heavy-chain antibodies, (ii) somatic hypermutation of amino acids in the heavy-chain antibodies (red asterisks), (iii) selection of B cells expressing higher affinity antibodies, and (iv) differentiation of specific B cells into antibody-secreting plasma cells (PC). The latter emigrate from the lymph node and migrate *via* the vascular system to the bone marrow, where some of these cells settle in long-lasting niches. **(C)** Explanation of the logos used in the diagrams to depict cell surface proteins.

Swelling of the draining lymph node and serum titers of specific antibodies can both be used as criteria for a successful immune response. Following ballistic DNA immunization, these responses typically are lower than with adjuvant-assisted protein or cell immunization, presumably owing to the additional time required for transcription, translation and posttranslational modifications and the longer lasting antigen exposure due to extended production of the antigen by long-living cells. In order to induce a shorter and stronger response, we typically perform a final boost with protein or cells. Specific Nbs can be selected with cDNA immunization alone and even in cases without detectable lymph node swelling and/or serum titers. After clonal expansion, antigen-specific B cells are thought to migrate in distinct short waves *via* the blood to the bone marrow. The timing of lymphocyte sampling from blood, therefore, is based on educated guessing and typically lies in the range of 4–14 days after boost immunization. It is, therefore, prudent to take multiple samples. The optimal timing for sampling lymphocytes *via* a lymph node biopsy presumably corresponds to the time of maximal swelling. In principle, it should also be possible to harvest bone marrow, but we are not aware that anyone has performed this yet on camelids. For all practical purposes, it is easiest to obtain blood samples—100 ml samples can readily be harvested from the jugular vein of a llama, alpaca or dromedary. If the draining lymph node responds by swelling, the optimal time window for harvesting of blood cells corresponds to a few days following the day of maximum lymph node swelling. DNA immunization often induces a slower and more extended response than adjuvant-assisted immunizations with protein or with cells. This likely reflects the additional time required for transcription and translation of the target protein and possibly also the time required for cytolysis of transfected cells by cytotoxic T cells and transport of membrane protein containing vesicles to the draining lymph node. We usually harvest blood 4–14 days after the last boost immunizations. Blood lymphocytes are purified by density gradient centrifugation, RNA is extracted and transcribed into cDNA using standard techniques. Optionally, plasma cells can be enriched by cell sorting prior to RNA extraction, e.g., using secondary antibodies against cell surface IgG or, by panning on cells expressing the membrane protein of interest. The Nb-coding region is then PCR amplified using appropriate primer pairs (see Figure 6). The PCR amplified VHH repertoire is then subjected to next-generation sequencing and cloning into pro- and or eukaryotic expression vectors (see Cloning of the VHH Repertoire and Identification of Specific Binders).

Using this approach we have generated functional Nbs against two ecto-enzymes and a ligand-gated ion channel (20–22). In case of murine ARTC2, three of four Nb families obtained from a single llama blocked the enzymatic activity of ARTC2 (20). A fourth Nb recognizes a distinct epitope and does not affect the enzyme activity. In case of human CD38 only three of 22 Nbs selected from four immunized llamas inhibited enzyme activity (22). Two Nbs that recognize a distinct epitope enhanced enzymatic activity. The other Nbs show little, if any effect on enzyme activity. In case of human P2X7 one of three Nbs isolated from two immunized llamas blocked channel function (21). In case of mouse P2X7 three of 19 Nbs blocked and two Nbs potentiated ATP-induced gating of P2X7. For CD38, we immunized two llamas with a mixture of expression constructs for human and mouse CD38 and two llamas with the purified ecto-domain of CD38 (22). For P2X7, we immunized two llamas with a mixture of expression constructs for human and mouse P2X7, and two llamas with P2X7-transfected cells (21). We obtained specific Nbs from each immunized animal. From these examples, we could not detect any consistent fundamental differences in the properties of Nbs obtained from cDNA vs. protein/cell immunized animals. We conclude that it is also possible to generate Nbs by protein ecto-domain immunization in case of single span proteins with a soluble extracellular domain, such as CD38. P2X7 and CXCR4 are examples of membrane proteins that cannot be produced as soluble ecto-domains. In such cases, immunization with transfected cells can be a useful alternative strategy to cDNA immunization.

## Mechanism of Antibody Induction by Genetic Immunization

Many of the events governing activation, proliferation, and maturation of B cells expressing a complementary antibody to the membrane protein of interest expressed by host cells after cDNA immunization are well understood (39–45). Figure 6 provides a schematic overview of the proposed mechanisms of antibody induction by cDNA immunization of camelids. The cDNA is transcribed into mRNA, the mRNA is translated into protein, and the protein is modified posttranslationally by the machinery of the transfected host cell (Figure 6A). A fraction of the expressed protein is degraded by proteasomes into peptides that are translocated into the lumen of the ER, where fitting peptides are loaded onto MHCI proteins. Bound peptides are presented by MHCI molecules on the surface of transfected cells, rendering the cells susceptible to attack by cytotoxic T cells (46). APC expressing the membrane protein and cellular debris including exosomes from the lysed cells are transported to the draining lymph nodes where they can be bound and endocytosed specifically by B cells expressing a complementary cell surface B cell receptor (BCR; Figure 6B). Following uptake and endosomal processing of the membrane protein by B cells, fitting peptides are presented by MHCII molecules on the cell surface of the B cell, rendering the cell responsive for help by CD4+ helper T cells (Th). Immune complexes containing target-exposing exosomes and antigen-specific antibodies are bound and displayed by follicular dendritic cells (FDCs). Binding of B cells to Th and FDCs triggers clonal expansion of the antigen-specific B cells in germinal centers. Clonal expansion is accompanied by somatic hypermutation of the genetic region encoding the variable domains. B cells expressing a cell surface immunoglobulin with higher affinity to the membrane protein displayed by FDCs expand preferentially, while B cells expressing a non or weakly binding surface immunoglobulin die by apoptosis. This Darwinian selection procedure results in the successive expansion of B cells with better fitting BCRs.

## Cocktail cDNA Immunizations

It is entirely feasible and in many cases even advantageous to perform cDNA immunizations with a mixture of two or more cDNA expression vectors. We routinely immunize camelids with vectors encoding the mouse and human orthologs of the membrane protein of interest. This often “kills three flies with one clap,” i.e., yielding Nbs specific for (i) the mouse ortholog, (ii) the human ortholog, and (iii) sometimes even Nbs cross-reactive with both the mouse and human orthologs. It is important to understand that camelids usually express the camelid ortholog of the cell surface protein of interest. The immune system typically recognizes only those portions of the target protein that are distinct from the endogenous protein. Antibodies induced by immunization with a human protein typically recognize the human protein but not the ortholog of the immunized camelid (e.g., llama, alpaca or dromedary). In other words, the antibody response usually is “blind” to conserved epitopes.

Cocktail immunization also provides the opportunity to augment an antibody response by providing helper epitopes (see Figure 6). Indeed, it is possible to induce a specific antibody response even if the target protein differs from the endogenous ortholog by only a single amino acid substitution, as exemplified by the classic Thy1 (CD90) alloantigens (47). Inbred strains of mice express either Thy1.1 or Thy1.2. Immunization of a Thy1.1+ mouse with cells expressing Thy1.2 induces a potent Thy1.2-specific antibody response, but only if the cells used for immunization co-express other disparate alloantigens that provide helper determinants (48). As explained in the previous section, antibody responses are driven by antigen-specific Th. This T cell help depends on both a peptide that can be presented by the particular MHCII molecules expressed by the immunized animal and a corresponding specific TCR that has escaped thymic selection. In order to obtain T cell help, the B cell needs to display MHCII-bound peptides on the cell surface. The MHCII presented peptides need not be derived from the membrane protein itself. They can also be derived from proteins encoded by a co-delivered cDNA expression vector during cocktail immunization, provided that the co-delivered protein is taken up and processed by the B cell displaying a specific antibody directed against the membrane protein of interest. In other words, suitable peptides must be delivered to the B cell together with the membrane protein of interest. It is unlikely that naive B cells endocytose intact EC or Langerhans cells. More likely, B cells bind and endocytose membrane-bound vesicles derived from these cells. If one or more distinct membrane proteins are contained in such vesicles, they can serve as a source for MHCII bound peptides.

Similarly, peptides binding to MHCI molecules typically drive cytotoxic T cell responses against the cells transfected with the cDNA expression construct during immunization (49, 50). Such a cytotoxic T cell response may indirectly support an antibody response by enhancing the release of vesicles containing the membrane protein from transfected cells, which can then be transported to the draining lymph nodes, thereby increasing the chances for encounter with and uptake by an antigen-specific B cell. As in case of MHCII-binding peptides, MHCI-binding peptides presented to cytotoxic T cells need not be derived from the target membrane protein of interest. With cDNA cocktail immunizations such peptides can be provided by a co-transfected protein, e.g., an antibiotic resistance marker under control of a mammalian promoter. Mixtures of cDNA expression vectors against two different membrane proteins of interest may therefore increase the chances of providing suitable peptides for activation of helper and cytotoxic T cells.

## Cloning of the VHH Repertoire and Identification of Specific Binders

**Figure 7:**
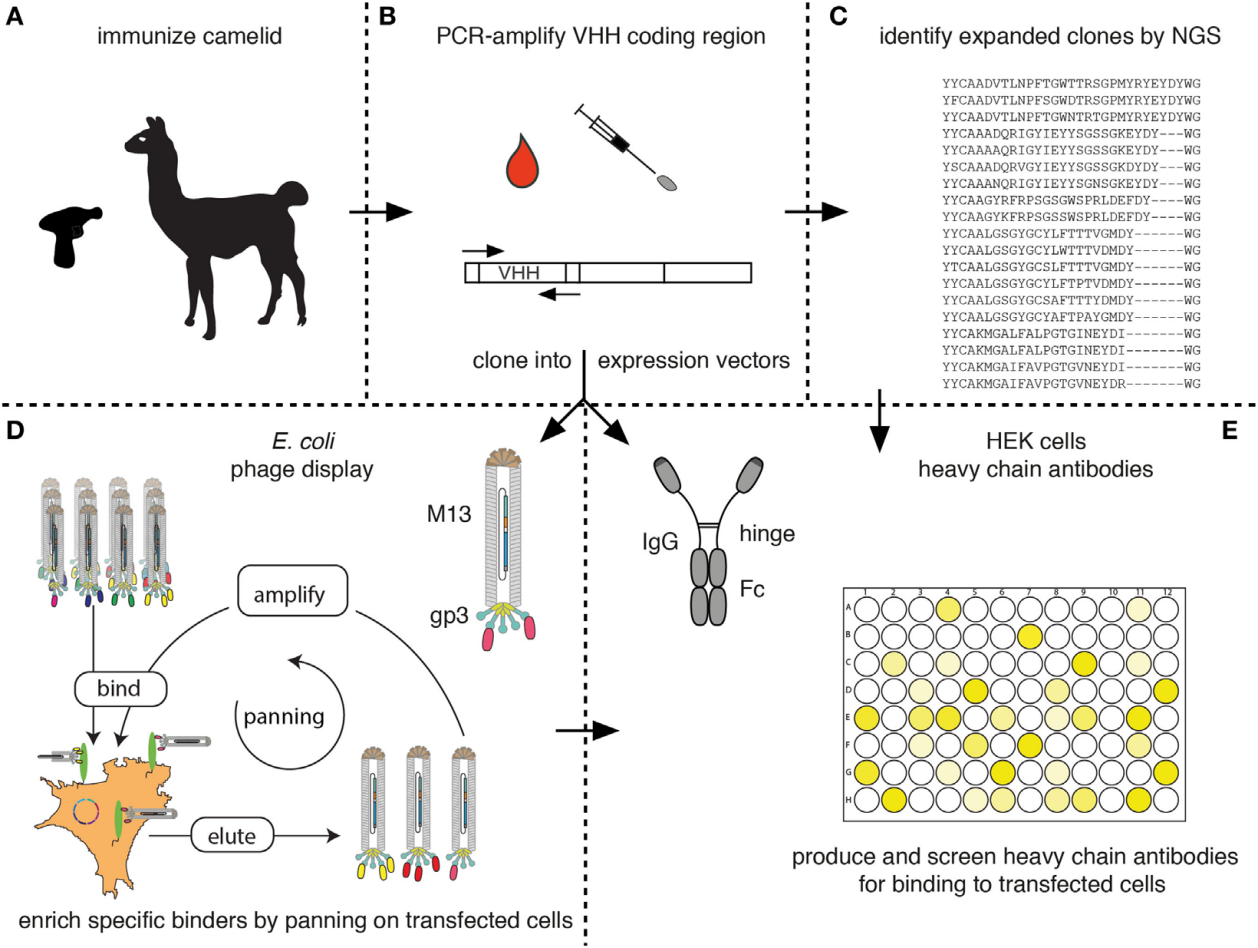
Schematic diagram for the identification of membrane protein-specific nanobodies (Nbs). This figure schematically illustrates the discovery path for membrane-protein-specific Nbs following cDNA immunization. **(A)** Camelids are immunized as described in Figure 5. **(B)** RNA is prepared from lymphocytes obtained from blood or a lymph node biopsy and transcribed into cDNA. The Nb (VHH) coding region is PCR amplified. **(C)** An aliquot of the PCR product is subjected to next-generation sequencing. The results permit identification of expanded clones and reveal the extent of somatic hypermutation in such clones. **(D,E)** A second aliquot of the PCR product is cloned into a bacterial **(D)** and/or mammalian **(E)** expression vector. **(D)** During cloning into the bacterial vector, the Nb is genetically fused to the gp3 capsid protein of the M13 phage. Antigen-specific clones can then be enriched from the phage display library by panning on cells transfected with the membrane protein of interest. Bound phage are eluted and panning can be repeated, e.g., on another cell type transfected with the protein of interest. Panning can be performed in the presence of available antibodies or Nbs in order to select for Nbs binding to a distinct epitope. **(E)** During cloning into the mammalian vector, the Nb is genetically fused to the hinge and Fc domains of a conventional rabbit, mouse, or human IgG. Individual clones are transfected into HEK cells. Heavy-chain antibodies secreted into the HEK cell supernatant are then screened for binding to cells transfected with the membrane protein of interest by ELISA or immunofluorescence microscopy using appropriate enzyme- or fluorochrome-conjugated secondary antibodies.

Figure 7 schematically outlines the procedure for cloning and sequencing of specific Nbs from immunized camelids. RNA extracted from blood lymphocytes (Figure 7A) is transcribed into cDNA and the Nb-coding region is then PCR amplified using appropriate primer pairs (Figure 7B). The-PCR-amplified VHH repertoire is then subjected to next-generation sequencing (Figure 7C) and cloning into pro- and or eukaryotic expression vectors (Figures 7D,E, respectively).

The fact that camelid heavy-chain antibodies recognize their target antigen *via* a single immunoglobulin domain provides a huge technical advantage for cloning of specific binders in comparison to conventional antibodies that recognize their target antigen via a pair of variable domains derived from two distinct protein chains. Cloning of the binding module from heavy-chain antibodies does not face the pairing problem of conventional antibodies. In other words, the VHH-domain can be PCR amplified and cloned directly into a mammalian expression vector so as to fuse the Nb to the hinge and Fc domains of a conventional mouse, human, or rabbit antibody (Figure 7E). It is then feasible to express such Nb-based heavy-chain antibodies in transiently transfected HEK cells, e.g., in a 96-well format and to screen the supernatants for production of specific binders. A second important advantage over conventional antibodies is that the entire ~350 bp coding sequence of each Nb is readily determined by a single sequence run. It can, thus, easily be determined whether identified binders are derived from the same or from distinct Nb families.

Next-generation sequencing of the PCR-amplified VHH repertoire (Figure 7C) provides information about expanded clones and about the degree of somatic hypermutation in expanded clones (12, 51, 52). If the samples are prepared from an appropriate source, e.g., a lymph node biopsy at the peak of B-cell clonal expansion or a sample of blood at the peak of migration of expanded clones from the lymph node to the bone marrow, most of the expanded clones are detected by NGS contain specific binders against the target protein of interest. Therefore, the results of next-generation sequencing can be used as a guide to specifically clone and express members of the expanded clones by simply ordering the coding sequence for each Nb as a synthetic DNA fragment and cloning this into a suitable mammalian heavy-chain antibody expression vector (Figures 7C–E).

A library of phage displayed Nbs can be used to select specific binders by panning of the library on immobilized antigen (Figure 7D). For membrane proteins, selection can be performed on cells transiently or permanently transfected with the protein of interest. In order to avoid selection of Nbs binding to irrelevant target proteins displayed on the surface of the cells used for selection, additional rounds of selection can be performed while changing the cell background. This can be achieved by using distinct cell types transfected with the same membrane protein of interest, e.g., human HEK cells in the first round and hamster CHO cells in the second round. Moreover, negative selections can be performed to remove phage binding to other cell surface proteins, using cells not expressing the protein of interest, e.g., untransfected HEK or CHO cells.

**Figure 8:**
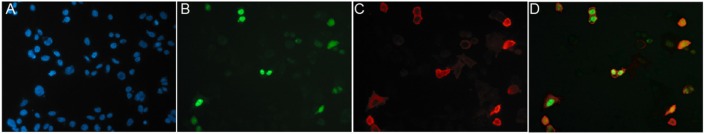
Identification of specific binders using CHO cells transiently co-transfected with expression vectors for the membrane protein of interest and nuclear GFP. CHO cells were transiently co-transfected with expression vectors for GFP fused to a nuclear localization sequence and the cDNA expression vector used for immunization, in this case encoding the homotrimeric ligand-gated P2X7 ion channel. Cells plated onto 96-well plates were analyzed 24 h after transfection by immunofluorescence microscopy. Green nuclei clearly distinguish transfected CHO cells from untransfectd CHO cells (blue nuclei stained with the DNA-binding dye Hoechst 33342). Cells were screened for cell surface binding of specific antibodies and bound antibodies were detected with a phycoerythrin (PE)-conjugated secondary antibody. **(A)** Hoechst dye, **(B)** GFP, **(C)** PE, and **(D)** merged images. Reprinted from Cellular Immunology 236, Sahil Adriouch, Gudrun Dubberke, Philip Diessenbacher, François Rassendren, Michel Seman, Friedrich Haag, Friedrich Koch-Nolte, Probing the expression and function of the P2X7 purinoceptor with antibodies raised by genetic immunization, 72–77, ©2005, with permission from Elsevier.

A convenient strategy for identifying binders using CHO cells transiently transfected with the same plasmid that was used for immunization is illustrated in Figure 8. We typically co-transfect CHO cells with expression vectors encoding the membrane protein of interest and a green fluorescent protein (GFP) fused to a nuclear localization signal (Figure 8). 24–48 h after transfection, the 10–20% of co-transfected cells are distinguished from untransfected cells by their green fluorescent nucleus. Nb-based rabbit IgG heavy-chain antibodies that bind specifically to co-transfected cells can readily be identified using a fluorochrome-conjugated anti-rabbit IgG secondary antibody.

## Assessing the Specificity, Affinity, and Relative Binding Epitopes of Target-Specific Nbs

The immunofluorescence assay described in Figure 8 also provides a convenient means to assess the specificity of the selected Nbs, i.e., by comparing the reactivity of the Nbs with CHO cells transfected with related membrane proteins. This provides information on the cross reactivity of the selected Nbs with orthologs of the membrane protein of interest from other species (e.g., human, mouse, rat) or with paralogs of the same species. The same assay can also be used to assess the relative affinity of the selected Nbs, i.e., by performing titration analyses.

Similarly, cross-blocking assays can provide information about the relative binding epitopes of different Nbs directed against the same membrane protein. To this end, cells are incubated sequentially with a large excess of one Nb before addition of the second Nb. The two Nbs must be distinguishable, e.g., *via* linkage to a moiety that permits independent detection of at least the second Nb. There are several options to achieve this, e.g., conjugation of the purified Nb-based heavy-chain antibody to biotin or a fluorochrome. Alternatively, the Nbs can be fused to two different IgG isotypes, e.g., rabbit IgG and human IgG1 allowing detection by isotype-specific secondary antibodies. The same procedure can be used to asses whether the selected Nbs bind to distinct or overlapping epitopes with commercially available monoclonal antibodies against the same target.

## Genetic Immunization to Raise Nbs Against Secretory and Intracellular Proteins

The strategy described here for raising Nbs against membrane proteins in native conformation can, in principle, be adapted also for raising Nbs against secretory or intracellular proteins in native conformation. Most secretory proteins can readily be produced as recombinant proteins and can thus also be used directly for classic adjuvant-assisted protein immunizations. For panning of phage libraries on secretory proteins, the target protein can be coated onto the walls of a standard 96-well ELISA plate or—after biotinylation—be captured on streptavidin-coated beads. Screening for specific binding can conveniently be performed on transiently transfected cells. In this case, signals can be enhanced by treatment of cells with Brefeldin A for 4–6 h prior to analysis in order to trap secretory proteins in the ER and/or Golgi apparatus. Moreover, cells need to be fixed and permeabilized in order for Nbs or Nb-based heavy-chain antibodies to access the Golgi and ER compartments.

cDNA immunization can effectively induce specific antibody responses also against intracellular proteins, e.g., GFP or neomycin phosphotransferase (our own unpublished observations), even though these intracellular proteins are not directly accessible to the BCR on the B cell plasma membrane. Presumably, cytolysis mediated by specific cytotoxic T cells results in the release of intracellular proteins and their transport to the B cell compartment in draining lymph nodes *via* the lymphatics. We have found that antibody responses against intracellular proteins can be enhanced by forced expression of the entire proteins or distinct domains thereof on the cell surface. To this end, the open reading frame for the entire intracellular protein or for a structurally independent subdomain of the protein is genetically fused upstream to an ORF encoding a signal peptide and downstream to an ORF encoding a GPI-anchor signal sequence. In many cases, this results in forced display of the protein or protein domain on the cell surface. However, not all intracellular proteins or protein domains fold properly in the oxidative environment of the ER. Moreover, if the protein (domain) contains an internal glycosylation site (e.g., N X S/T), it can be aberrantly glycosylated in the ER. Furthermore, unpaired cysteine residues can engage in disulfide bond formation in the ER. These problems can often be circumvented by site-directed mutagenesis, e.g., by conservative substitutions such as asn to gln, or cys to ser. It may be convenient to attach an epitope tag—e.g., a FLAG tag to the N-terminus of the protein (domain), so that successful cell surface expression can be monitored by flow cytometry or immunofluorescence microscopy with a tag-specific antibody. If successful, immunization and selection of Nbs then follow the procedures described above for membrane proteins.

## Advantages and Limitations of Ballistic cDNA Immunization

Key advantages of the cDNA immunization approach are the high purity of the immunogen and the presentation of the target protein in its native conformation. Moreover, this approach offers the opportunity to co-immunize with other proteins of interest, e.g., target ortholog(s) of other species, a foreign protein(s) as a source of MHC-binding peptides, and inflammation-promoting cytokines. A limitation of cDNA immunization vs. adjuvant-assisted protein or cellular immunizations is the prolonged induction of the immune response resulting from the time required for transcription, translation, and posttranslational modifications. Moreover, lymph node swelling and serum titer typically are lower with cDNA than with protein immunizations. Consequently, it is more difficult to judge the optimal timing of lymphocyte sampling. Another limitation is the high cost of the gene gun. A recent report describes the successful selection of ChemR23-specific Nbs (a GPCR) upon immunization of llamas with the dermojet (AKRA DERMOJET), a needle-less injection device (34). Nbs elicited by cDNA immunization do not differ in terms of affinity, specificity, or other fundamental properties from Nbs elicited by alternative approaches, such as liposomes or virus-like particles bearing membrane proteins.

## Conclusion and Perspectives

Nanobodies and Nb-based heavy-chain antibodies have already proved valuable experimental tools in infection, immunity, oncology, and numerous other settings. The first Nb expected to be licensed for clinical applications next year (caplacizumab) is directed against a soluble protein (von Willebrand factor). This will likely pave the way for a wave of new Nb-based reagents to enter the clinic. Membrane proteins expressed by immune cells and tumor cells in particular represent potential therapeutic Nb targets in immunology and oncology. Conventional immunization strategies with purified protein and/or transfected cells are hampered by the poor solubility of many membrane proteins outside of a lipid environment and by the multitude of other antigens in transfected cells. The cDNA immunization strategy described here provides a powerful tool for the development of Nbs directed against membrane proteins.

## Author Contributions

FH and FK-N conceived the project. All authors established experimental procedures. TE, SM, JW, and FK-N wrote the manuscript. All authors reviewed and approved the manuscript.

## Conflict of Interest Statement

FH and FK-N receive a share of antibody sales *via* MediGate GmbH, a wholly owned subsidiary of the University Medical Center Hamburg-Eppendorf. TE, SM, JW, and FK-N are co-inventors on patent applications on nanobody transgenic mice and/or CD38- or P2X7-specific nanobodies. The reviewer GH and handling editor declared their shared affiliation.
